# Editorial: Regulation of plant immunity by immune receptors

**DOI:** 10.3389/fpls.2023.1320509

**Published:** 2023-10-26

**Authors:** Wei Wang, Zhaoyang Zhou, Ali Noman, Yasuhiro Kadota

**Affiliations:** ^1^ State Key Laboratory of Ecological Control of Fujian-Taiwan Crop Pests, Key Laboratory of Ministry of Education for Genetics, Breeding and Multiple Utilization of Crops, Plant Immunity Center, Fujian Agriculture and Forestry University, Fuzhou, China; ^2^ Ministerial and Provincial Joint Innovation Centre for Safety Production of Cross-Strait Crops, Fujian Agriculture and Forestry University, Fuzhou, China; ^3^ Beijing Key Laboratory of Growth and Developmental Regulation for Protected Vegetable Crops, Department of Vegetable Sciences, China Agricultural University, Beijing, China; ^4^ Department of Botany, Government College University, Faisalabad, Pakistan; ^5^ RIKEN Center for Sustainable Resource Science (CSRS), Plant Immunity Research Group, Yokohama, Japan

**Keywords:** plant immunity, PRRs, NLRs, MAMPs, effectors, plant-pathogen interaction

In nature, one of the largest challenges of plants is to cope with multiple pathogens. Unlike animals, plants lack an adaptive immune system. To defend against pathogen attacks, plants employ cell surface-localized and intracellular immune receptors to activate immunity (Jones and Dangl, 2006). Cell surface-localized pattern-recognition receptors (PRRs), belonging to receptor-like kinase (RLK) or receptor-like protein (RLP) families, sense conserved microbe-associated molecular patterns (MAMPs) or damage-associated molecular patterns (DAMPs) to trigger immune responses in plants (Tang et al., 2017; Wang et al., 2020). Intracellular nucleotide binding leucine-rich repeat domain-containing receptors (NLRs) recognize cytoplasmic effectors secreted by pathogens to trigger plant defenses (Monteiro and Nishimura, 2018; Zhou and Zhang, 2020). Both PRRs and NLRs are immune receptors. Recently, several studies demonstrated that NLR-activated immune responses enhance the expression and abundance of PRRs, and PRR-initiated immune signals contribute to the complete activation of NLR-mediated immunity (Ngou et al., 2021; Pruitt et al., 2021; Tian et al., 2021; Yuan et al., 2021). However, the underlying mechanism by which immune receptors regulate plant resistance is still largely unclear.

This Research Topic consists of 4 research articles and 2 review articles addressing the mechanism of plant defense responses mediated by immune receptors. PRRs usually contain ectodomains, such as leucine-rich repeats (LRRs), lysine motifs (LysMs), lectin domains, or epidermal growth factor (EGF)-like domains, that perceive different patterns (Saijo et al., 2018; Bender and Zipfel, 2023). In Arabidopsis, LYK5, a LysM receptor-like kinase (RLK), has been reported to associate with LYK4 and CHITIN ELICITOR RECEPTOR KINASE 1 (CERK1) to activate immune responses upon perceiving chitin, the major component of fungal cell walls, or its deacetylated derivative chitooligosaccharides (COS) (Wan et al., 2012; Cao et al., 2014; Xue et al., 2019). In grapevine (*Vitis vinifera*), two PRRs, VvLYK1-1 and VvLYK1-2, have been identified to sense COS (Brule et al., 2019; Heloir et al., 2019). Roudaire et al. highlights that heterologous expression of *VvLYK5-1* and *VvLYK5-2* can rescue the COS sensitivity of the *atlyk4/5* mutant in Arabidopsis. Moreover, VvLYK5-1 interacts with VvLYK1-1 after the perception of chitin, indicating that VvLYK5-1 and VvLYK5-2 are two additional receptors that perceive chitin in grapevine. Lectin receptor-like kinases (LecRKs) are widely distributed in plant kingdoms. Liu et al. summarizes the most recent advances in ligand recognition of LecRKs, which presents a panoramic view of how LecRKs recognize their ligands and how posttranslational modifications contribute to LecRK-mediated plant immunity. Phytocytokine peptides have regulatory functions in the control of plant immune responses. RAPID ALKALINIZATION FACTORs (RALFs) are cysteine-rich peptides generated and secreted by plants. A more recent study demonstrated that RALF22 enhances immune signals induced by the plant endogenous peptide pep3 (He et al., 2023). Mamaeva et al. investigates the role of RALFs in the coordination of plant growth and stress response in the nonvascular moss *Physcomitrium patens*. PpRALF2 and PpRALF3 positively regulate plant growth but negatively regulate resistance to multiple pathogens, suggesting that RALF peptides may be involved in the trade-off of growth and defense.

NLR proteins play a critical role in recognizing pathogen-secreted cytoplasmic effectors and activating resistance. Many activated NLRs have been found to form a plasma membrane-associated pore-like resistosome, which acts as a cation channel (Wang et al., 2019a; Wang et al., 2019b; Martin et al., 2020; Bi et al., 2021; Jacob et al., 2021). However, some NLR proteins need to pair with another NLR to initiate plant immunity. These genes encoding paired NLRs often link in a head-to-head configuration. Moreover, some of the paired NLRs usually carry an integrated domain (ID), such as a WRKY domain and a heavy metal-associated (HMA) domain, which interacts with pathogen effectors (Cesari et al., 2014; Williams et al., 2014; Monteiro and Nishimura, 2018). Guo et al. summarizes the molecular basis of HMA ID from paired NLRs revealed by structural and biochemical studies, according which the HMA ID-based design of rice materials with broad-spectrum resistance possesses great application. The high expression of *NLR* genes may be a disadvantage to plant growth. von Dahlen et al. analyzes the expression of 940 *NLR* genes of tomato and potato across 315 transcriptome libraries. Most *NLR* genes are expressed at a low level but are induced during infection under tissue-specific transcriptional regulation. Remarkably, Li et al. presents a thorough analysis of *NLR* genes across 100 high-quality plant genomes (PlantNLRatlas). This database supports a comprehensive analysis of *NLR* genes across a diverse array of species and holds the potential value to deeply understand the evolution, structure, and function of NLRs.

Immune receptors are crucial for plant resistance; therefore, the study of immune receptor activation and regulation in plant immunity is one of the most active fields in plant research. The underlying mechanisms by which plants regulate immunity through immune receptors are being investigated. We hope that this Research Topic will help readers to have a better understanding of the advances that have been made recently in plant immunity, which will facilitate the development of engineering elite crops.

## Author contributions

WW: Writing – original draft, Writing – review & editing. ZZ: Writing – review & editing. AN: Writing – review & editing. YK: Writing – review & editing.
